# *ESR1* fusions drive endocrine therapy resistance and metastasis in breast cancer

**DOI:** 10.1080/23723556.2018.1526005

**Published:** 2018-10-09

**Authors:** Jonathan T. Lei, Xuxu Gou, Matthew J. Ellis

**Affiliations:** a Interdepartmental Graduate Program in Translational Biology & Molecular Medicine, Baylor College of Medicine, Houston, TX, USA; b Lester and Sue Smith Breast Center, Baylor College of Medicine, Houston, TX, USA; c Departments of Medicine and Molecular and Cellular Biology, Baylor College of Medicine, Houston, TX, USA

**Keywords:** *ESR1* fusions, breast cancer, endocrine therapy resistance, EMT, metastasis

## Abstract

Estrogen receptor alpha gene (*ESR1*) fusion transcripts have been identified in breast cancer but their role in breast cancer is not completely understood. Here, we report a causal role for *ESR1* fusions in driving both endocrine therapy resistance and metastasis, and describe a therapeutic strategy to target *ESR1* fusion-induced growth.

The majority of breast cancers express the nuclear hormone receptor, estrogen receptor alpha (ER) and therefore are fueled by estrogen and downstream ER signaling. Despite the tremendous success endocrine therapies, resistance and the development of lethal metastatic disease is common and a major clinical problem.

Dysregulation of the estrogen receptor alpha gene (*ESR1*) is an established mechanism of inducing endocrine therapy resistance in ER positive (ER+) breast cancer. Recurrent point mutations clustering around the ligand binding domain (LBD) of *ESR1* that cause single amino acid residue changes have been found in up to 40% of treatment-refractory, metastatic ER+ breast cancer patients (reviewed in ^1^). These activating *ESR1* point mutations confer constitutive, hormone-independent activity of ER and are often enriched in breast tumors after aromatase inhibitor (AI) treatment.

Emerging evidence now suggests genomic rearrangement events involving *ESR1* producing *ESR1* fusion genes are another class of somatic mutation that is associated with endocrine therapy resistance. One class of recurrent *ESR1* fusion transcripts have been found in a subset of Luminal B primary breast tumors that retain the first two non-coding exons of *ESR1* (ESR1-e2) fused to various sequences from a nearby gene, coiled-coil domain containing 170, *CCDC170* (ESR1-e2>CCDC170), potentially generated by tandem-duplication, based on the observed orientation of the fusion sequences.^2^ This fusion gene forms a promoter trap driving aberrant expression of *CCDC170* since this gene is now in the context of the *ESR1* promoter, producing stable truncated forms of CCDC170 protein (ΔCCDC170). Specific forms of ΔCCDC170 leads to reduced tamoxifen sensitivity in experimental models.^2^ Recently, an additional ESR1-e2 fusion with the acidic residue methyltransferase 1 gene, *C6orf211* (ESR1-e2>C6orf211) fusion as well as ESR1-e2>CCDC170 fusions have been identified in AI resistant breast tumors, but more studies are required to test whether these ESR1-e2 fusions produce pathogenic proteins.^3^ Taken together, these *ESR1* fusion events have the potential to generate pathogenic, truncated forms of fusion partner proteins, but does not produce *ESR1* fusion proteins (Figure 1B).

**Figure 1.**
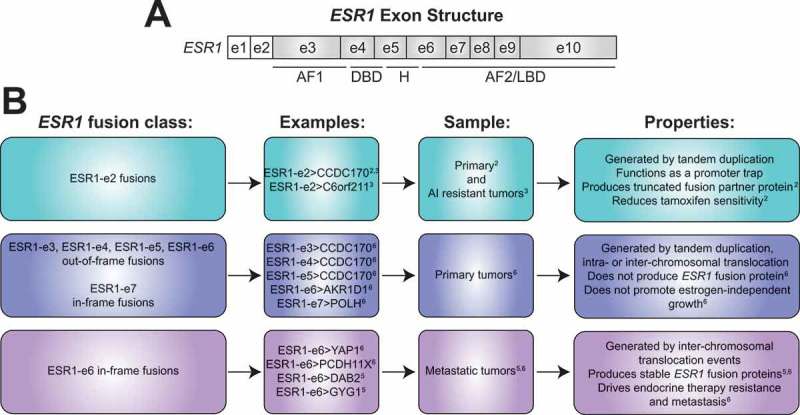
**Landscape of *ESR1* fusions in estrogen receptor positive breast cancer. (A)** Structure of the estrogen receptor alpha gene (*ESR1*) containing 10 exons (e). The first two non shaded boxes represent non-coding exons (e1 and e2) followed by shaded boxes representing exons encoding for activation function 1 domain (AF1), DNA-binding domain (DBD), hinge region (H), and the activation function 2 (AF2)/ligand binding domain (LBD). **(B)** Three major classes of *ESR1* fusion transcripts identified in estrogen receptor positive breast tumors. The first class arises from tandem duplication of *ESR1*promoter sequences including the first two exons of *ESR1* (ESR1-e2) and a nearby gene found in primary and aromatase inhibitor (AI) resistant tumors, producing truncated forms of fusion partner protein, and therefore potentially driving disease pathogenesis by functioning as a promoter trap. The second class consists of the first 3–6 exons of *ESR1* (ESR1-e3, ESR1-e4, ESR1-e5, ESR1-e6) fused out-of-frame or the first 7 exons of *ESR1* (ESR1-e7) fused in-frame to C-terminal sequences from the partner gene. These fusion genes are found in treatment naïve primary breast tumors and do not produce stable *ESR1* fusion proteins nor induce growth in estrogen-deprived conditions. The third class involves the first 6 exons of *ESR1* (ESR1-e6) fused in-frame to C-terminal partner gene sequences provided by inter-chromosomal translocation found in metastatic breast tumors that have been extensively pretreated with endocrine agents. ESR1-e6>YAP1 and ESR1-e6>PCDH11X have been functionally characterized in detail and have been shown to drive endocrine therapy resistance and metastasis in experimental models. Coiled-coil domain containing protein 170, *CCDC170*; acidic residue methyltransferase 1, *C6orf211*; aldo-keto reductase family 1 member D1, *AKR1D1*; DNA polymerase eta, *POLH*; yes associated protein 1, *YAP1*; protocadherin 11 X-linked, *PCDH11X*; disabled homolog 2, *DAB2*; glycogenin 1, *GYG1.*

Another mechanism that can produce *ESR1* fusions is inter- or intra-chromosomal translocation events where the *ESR1* gene is fused to more distant locations in the genome. Our group described the first stable and functional *ESR1* fusion protein produced by a fusion gene involving the first six exons of *ESR1* (ESR1-e6) fused in-frame to C-terminal sequences of the yes associated protein 1 gene, *YAP1* (ESR1-e6>YAP1) provided by an inter-chromosomal translocation event.^4^ This was identified in a patient with endocrine therapy-resistant, metastatic ER+ disease and in a matched patient-derived xenograft (PDX). A recent study described additional ESR1-e6 in-frame, inter-chromosomal translocation events involving the disabled homolog 2 gene, *DAB2*, and the glycogenin 1 gene, *GYG1* (ESR1-e6>DAB2 and ESR1-e6>GYG1) producing stable in-frame *ESR1* fusion proteins at metastatic sites in endocrine therapy refractory ER+ breast cancer patients.^5^ All of these ESR1-e6 fusions retain the N-terminus of *ESR1* encoding the DNA binding and nuclear localization domains, suggesting some functionality. However, detailed functional characterization and studies demonstrating a causal role for *ESR1* fusions in endocrine therapy resistance and metastasis has been lacking (Figure 1B).

Our current study investigated the role of *ESR1* fusion genes in driving therapeutic resistance and metastasis in ER+ breast cancer.^6^ We identified a variety of in-frame and out-of-frame translocations involving *ESR1* from RNA-seq analysis of primary and metastatic ER+ breast samples. In-frame fusions included inter-chromosomal *ESR1* translocations with the *YAP1* gene (ESR1-e6>YAP1) as previously described,^4^ the protocadherin 11 X-linked gene, *PCDH11X* (ESR1-e6>PCDH11X) and the nucleolar protein 2 homolog gene, *NOP2* (ESR1-e6>NOP2), and two intra-chromosomal translocations with the A-kinase anchoring protein 12 gene, *AKAP12* (ESR1-e6>AKAP12) and the DNA polymerase eta gene, *POLH* (ESR1-e7>POLH). However, only inter-chromosomal *ESR1* fusions, ESR1-e6>YAP1, ESR1-e6>PCDH11X, and ESR1-e6>NOP2, produced stable in-frame *ESR1* fusion proteins *in vitro*. Of these, the two *ESR1* fusions identified from endocrine therapy-refractory, ER+ metastatic disease, ESR1-e6>YAP1 and ESR1-e6>PCDH11X, drove endocrine therapy resistant proliferation in experimental models, while ESR1-e6>NOP2 from an endocrine therapy naïve primary tumor did not. In addition, none of the out-of-frame ESR1-e3, ESR1-e4, ESR1-e5, and ESR1-e6 containing fusions nor even an in-frame ESR1-e7 fusion, all identified in primary tumors, were able to drive growth under estrogen-deprived conditions (Figure 1B).

To explore transcriptional properties of the *ESR1* fusions that produced stable *ESR1* fusion proteins, chromatin immunoprecipitation followed by next-generation sequencing (ChIP-seq) was performed along with RNA-seq in T47D cell lines to examine regulation of *ESR1* fusion bound genes. The ESR1-e6>YAP1 and ESR1-e6>PCDH11X drove constitutive expression of these ER target genes in the absence of estrogen, demonstrating strong estrogen-independent transcriptional activation. The ESR1-e6>NOP2 bound relatively few sites compared to the other *ESR1* fusions, potentially explaining the weak functional activity in proliferation assays. Interestingly, a cluster of genes was found to be strongly and uniquely up-regulated by the ESR1-e6>YAP1 and ESR1-e6>PCDH11X. Pathway analysis revealed enrichment of epithelial-to-mesenchymal transition (EMT) genes, including induction of an established EMT gene, snail family transcriptional repressor 1, *SNAI1*. Subsequent functional studies showed the transcriptionally active ESR1-e6>YAP1 and ESR1-e6>PCDH11X fusions induced cell motility *in vitro* and drove metastasis to the lung in xenograft models.

Since the formation of these *ESR1* fusion proteins lacks the LBD of *ESR1*, all known endocrine therapies that target the LBD are likely to be ineffective. Therefore, we targeted downstream ER signaling events by using a United States Food and Drug Administration approved cyclin-dependent kinase (CDK) 4/6 inhibitor for metastatic ER+ breast cancer, palbociclib, based on our previous observation that palbociclib antagonized tumor growth driven by *ESR1* point activating mutations. Palbociclib suppressed growth driven by ESR1-e6>YAP1 and ESR1-e6>PCDH11X *in vitro*, and at primary and metastatic sites in a PDX model naturally harboring the ESR1-e6>YAP1 fusion.

Taken together, these results further our understanding of the mechanisms underlying endocrine therapy resistance and metastasis in ER+ breast cancer. Transcriptionally active in-frame ESR1-e6 fusions such as ESR1-e6>YAP1 and ESR1-e6>PCDH11X constitutively drives expression of not only ER target genes leading to endocrine therapy-resistant proliferation but also induces EMT genes leading to metastasis (Figure 1B). Therefore, formation of *ESR1* fusion genes links these two processes together, potentially explaining the lethal outcomes in the patients these fusions were identified.

These findings also have important therapeutic and diagnostic implications. Since *ESR1* fusion driven growth remained sensitive to CDK4/6 inhibition, the presence of an in-frame *ESR1* fusion could be used as a biomarker to stratify patients for CDK4/6 inhibitor therapy. Also, a pattern of *ESR1* fusions is emerging in metastatic ER+ breast cancer, in which the first six exons of *ESR1* are fused in-frame to C-terminal partner sequences provided by the partner gene. This finding could potentially drive targeted sequencing approaches to efficiently identify additional active *ESR1* fusions by using a 3ʹ exon sequence against *ESR1* exon 6 as bait.

In conclusion, *ESR1* fusions are a new class of recurrent somatic mutations that drive endocrine therapy resistance and metastasis in ER+ breast cancer. This study adds to the catalog of actionable *ESR1* alterations and furthers our understanding of how ER+ breast cancer gives rise to lethal metastatic disease.
